# Supplementary material for: Deficiency in Androgen Receptor Aggravates the Depressive-Like Behaviors in Chronic Mild Stress Model of Depression
Source: Cells. 2019 Sep 2;8(9):1021. doi: 10.3390/cells8091021 (PMC6769639; doi:10.3390/cells8091021)
Supplement: Supplementary file 1 [file cells-08-01021-s001.pdf]

	MDD patients n = 21	Health controls n = 15	P value
Age (year)	44.86 ± 8.66	39.80 ± 15.72	0.170
BMI (kg/m^2)	22.63 ± 3.76	23.71 ± 3.35	0.391
HAMD-17 score	28.55	-	-
AR mRNA (-Delta CT)	-11.69 ± 1.37	-10.81 ± 1.04	0.042*

Supplementary Table S1. AR mRNA expression level is lower in PBMCs from male patients with major depressive disorder (MDD) compared with male healthy controls. BMI=body mass Index, HAMD=the 17-item Hamilton Depression Rating Scale.

*** Mann-Whitney U test**
